# Therapeutic Potential of Peroxisome Proliferator-Activated Receptor (PPAR) Agonists in Substance Use Disorders: A Synthesis of Preclinical and Human Evidence

**DOI:** 10.3390/cells9051196

**Published:** 2020-05-12

**Authors:** Justin Matheson, Bernard Le Foll

**Affiliations:** 1 Department of Pharmacology and Toxicology, Faculty of Medicine, University of Toronto, 27 King’s College Circle, Toronto, ON M5S 3H7, Canada; bernard.lefoll@camh.ca; 2 Institute for Mental Health Policy Research, Centre for Addiction and Mental Health, 33 Russell Street, Toronto, ON M5S 2S1, Canada; 3 Translational Addiction Research Laboratory, Centre for Addiction and Mental Health, 33 Russell Street, Toronto, ON M5S 2S1, Canada; 4 Addictions Division, Centre for Addiction and Mental Health, 100 Stokes Street, Toronto, ON M6J 1H4, Canada; 5 Campbell Family Mental Health Research Institute, Centre for Addiction and Mental Health, 250 College Street, Toronto, ON M5T 1R8, Canada; 6 Department of Psychiatry, Faculty of Medicine, University of Toronto, 250 College Street, Toronto, ON M5T 1R8, Canada; 7 Institute of Medical Sciences, University of Toronto, 1 King’s College Circle, Room 2374, Toronto, ON M5S 1A8, Canada; 8 Department of Family and Community Medicine, University of Toronto, 500 University Avenue, 5th Floor, Toronto, ON M5G 1V7, Canada

**Keywords:** PPAR, nuclear receptors, addiction, alcohol, nicotine, opioids, psychostimulants, animal models, human studies

## Abstract

Targeting peroxisome proliferator-activated receptors (PPARs) has received increasing interest as a potential strategy to treat substance use disorders due to the localization of PPARs in addiction-related brain regions and the ability of PPAR ligands to modulate dopamine neurotransmission. Robust evidence from animal models suggests that agonists at both the PPAR-α and PPAR-γ isoforms can reduce both positive and negative reinforcing properties of ethanol, nicotine, opioids, and possibly psychostimulants. A reduction in the voluntary consumption of ethanol following treatment with PPAR agonists seems to be the most consistent finding. However, the human evidence is limited in scope and has so far been less promising. There have been no published human trials of PPAR agonists for treatment of alcohol use disorder, despite the compelling preclinical evidence. Two trials of PPAR-α agonists as potential smoking cessation drugs found no effect on nicotine-related outcomes. The PPAR-γ agonist pioglitazone showed some promise in reducing heroin, nicotine, and cocaine craving in two human laboratory studies and one pilot trial, yet other outcomes were unaffected. Potential explanations for the discordance between the animal and human evidence, such as the potency and selectivity of PPAR ligands and sex-related variability in PPAR physiology, are discussed.

## 1. Introduction

Substance use disorders (SUDs) continue to represent a significant global public health burden. In 2017, of the estimated 271 million people aged 16–64 years worldwide who had used drugs in the past year, nearly 35 million (~13%) were estimated to suffer from an SUD [1]. An SUD is a diagnostic entity in the Diagnostic and Statistical Manual, 5th Edition (DSM-V) that refers to the repeated use of a substance that causes significant impairment, e.g., continued use despite physical and psychological harms and failure to meet expectations at work or school [2]. The term “addiction” is often used to refer to the severe stage of an SUD characterized by compulsive drug-seeking despite negative consequences [3,4] that runs a chronic, relapsing course with poor long-term durability of abstinence from drug-taking even with treatment [5].

Research into the neurobiology of addictions over the past few decades has substantively advanced our understanding of the key facets of compulsive drug-taking [6,7]. For example, while the focus of early addictions research was the acute, positively reinforcing properties of drugs of abuse, it is now recognized that negatively reinforcing states involving anhedonia, dysphoria, and anxiety become more important in maintaining drug-taking over time [7]. As a result, motivation to use the drug shifts from seeking pleasure to avoiding negative affect. Thus, pharmacotherapeutic strategies to treat addictions need to not only reduce the reinforcing or rewarding properties of drugs, but also target the negatively reinforcing states associated with chronic drug-taking that contribute to the significant risk of relapse [7]. Agonist substitution therapies have been successful in mitigating this negative reinforcement in some SUDs, e.g., methadone or buprenorphine for managing withdrawal and craving associated with opioid use disorder [8] and nicotine replacement therapy (NRT) for managing nicotine withdrawal [9]. Other medications, such as naltrexone or acamprosate for alcohol use disorder [10] and varenicline or bupropion for nicotine dependence [9], have demonstrated some efficacy in reducing positive and/or negative reinforcing aspects of drug use. Nevertheless, long-term abstinence rates remain low across SUDs, highlighting the need for novel pharmacological treatment approaches.

Peroxisome proliferator-activated receptors (PPARs) are a subfamily of nuclear receptors that dimerize with retinoid X receptors (RXRs) to regulate gene expression by binding to specific peroxisome proliferator response elements (PPREs) in enhancer sites of select genes [11]. Three isoforms of PPARs have been identified: α, γ, and β/δ. So far, the therapeutic potential of PPAR ligands had been in non-psychiatric fields. While PPARs were initially identified as lipid sensors [11], burgeoning evidence has demonstrated a role of these nuclear receptors in a wide range of physiological functions, including central nervous system (CNS) functions such as memory consolidation and modulation of pain perception [12]. PPAR agonists have been recently considered for their potential to treat neuropsychiatric disorders, largely due to their ability to target levels of neuroinflammation thought to be involved in the pathophysiology of these illnesses [13]. In particular, mounting evidence of an important relationship between neuroimmune function and addition-related processes has generated interest in investigating the role of PPARs in drug-related behaviors [14,15].

Converging lines of evidence have also suggested a more direct role of PPARs in addiction-relevant neurocircuitry. Initial evidence came from studies demonstrating that selective inhibition of fatty acid amide hydrolase (FAAH), an enzyme responsible for degradation of the endogenous cannabinoid anandamide and the endogenous PPAR ligands oleoylethanolamide (OEA) and palmitoylethanolamide (PEA), could suppress nicotine-induced activation of dopamine neurons in rats [16,17]. Importantly, this effect was mimicked by OEA and PEA, but not anandamide, suggesting the effect was due to PPAR activation specifically [16]. Exogenous PPAR agonists have also been demonstrated to attenuate nicotine-induced [18,19] and heroin-induced [20] excitation of dopamine neurons in the ventral tegmental area (VTA) and elevations of dopamine in the nucleus accumbens (NAc) shell in rats. Further confirmatory evidence comes from rodent studies demonstrating that PPAR isoforms are indeed localized in addiction-relevant brain regions such as the VTA [21,22], an important part of the mesocorticolimbic dopaminergic system that plays a central role in drug-related reward [7], and that PPAR-γ colocalizes with tyrosine-hydroxylase-positive cells in the VTA, suggesting direct expression in dopaminergic neurons [23]. A detailed presentation of the neurobiological substrates mediating the impact of PPAR agonists on addiction-related behaviors is beyond the topic of the present review (see [18,19] for some mechanistic studies).

The goal of the present review is to expand upon our previous review of the preclinical evidence for a role of PPARs in addiction [24] to incorporate novel preclinical findings as well as the current state of evidence from clinical and laboratory studies in humans.

## 2. Preclinical Behavioral Evidence

Evidence for the role of PPAR agonists in modifying addiction-like behaviors in animal models is broadly divided into two categories: drug consumption/motivation to use and withdrawal/relapse. A summary of key methodological details and relevant findings of the studies reviewed is provided in Table 1.

### 2.1. PPAR-α Agonists

#### 2.1.1. Consumption/Motivation

A significant body of evidence has consistently demonstrated that PPAR-α agonists can attenuate voluntary consumption and operant self-administration of ethanol in rodents [25,26,27,28,29,30,31,32]. Using the two-bottle choice paradigm, studies have found a decrease in voluntary consumption of ethanol following administration of the clinically useful drugs gemfibrozil [25] and fenofibrate [26,27,29,30,32], the endogenous agonist OEA [28], the experimental agonist WY14643 [28], and the dual PPAR-α/γ agonist tesaglitazar [26,29,30]. In addition, operant self-administration of ethanol was attenuated following administration of OEA and WY14643 under a one-response fixed ratio (i.e., FR1) schedule [28] and fenofibrate under FR2 and progressive ratio (PR) schedules [31]. Importantly, the effects of the PPAR-α agonists on attenuation of voluntary consumption of ethanol were reversed when animals were pre-treated with the PPAR-α antagonists GW6471 [28] or MK886 [30]. Overall, these results strongly support a role of PPAR-α agonism in reducing willingness to consume ethanol and in reducing the reinforcing properties of ethanol.

Two studies have assessed the effects of fenofibrate on the development of ethanol conditioned place preference (CPP) as a measure of the rewarding effects of ethanol, with mixed results [32,33]. Blednov et al. (2016) found no effect of oral administration of 150 mg/kg fenofibrate or 1.5 mg/kg of the dual PPAR-α/γ agonist tesaglitazar on the development of ethanol CPP in male mice [33]. However, Rivera-Meza et al. (2017) found that oral administration of 50 mg/kg fenofibrate attenuated the development of ethanol CPP in male rats selectively bred for high ethanol intake (i.e., UChB rats) [32]. The inconsistency between these two studies is unclear but could be due to the different doses of fenofibrate used or differences in ethanol-related behaviors of the two different animal models.

More limited, but robust, evidence has supported a role of PPAR-α agonists in attenuating operant self-administration of nicotine in rodents and non-human primates [18,19]. Mascia et al. (2011) found that both WY14643 and methyl-OEA reduced nicotine self-administration under an FR5 schedule in rats and an FR10 schedule in monkeys, and that these effects were reversed by co-administration of the PPAR-α antagonist MK886 [18]. WY14643 had no effect on operant self-administration of cocaine in monkeys, demonstrating specificity to nicotine [18]. Panlilio et al. (2012) found further evidence that the clinically useful drug clofibrate prevented the acquisition of self-administration in naïve rats and decreased self-administration in experienced rats and monkeys, an effect that was reversed by treatment with MK886 [19]. Neither study found an effect of PPAR-α agonists on nicotine discrimination [18,19].

Two additional studies have suggested a role of PPAR-α agonists in attenuating nicotine CPP [40,43]. Jackson et al. (2017) found that both WY14643 and fenofibrate significantly reduced nicotine preference in the CPP experiments, though fenofibrate was less potent [40]. Importantly, WY14643 did not shift the potency of nicotine in the CPP paradigm, and the effect of WY14643 was specific to nicotine as it had no effect on cocaine preference [40]. In support of these findings, Donvito et al. (2019) found that exogenous administration of the lipid transmitter n-Oleoyl-glycine (OlGly) prevented the development of nicotine, but not morphine, CPP, and that this effect was blocked by the PPAR-α antagonist GW6471 [43]. Taken together, the results of the operant self-administration and CPP experiments provide strong support for a role of PPAR-α agonism in reducing the reinforcing and rewarding properties of nicotine.

Finally, one study found that OEA reduced behavioral sensitization to cocaine and cocaine CPP, though this effect was intact in PPAR-α KO mice, suggesting this was due to a PPAR-independent mechanism [36].

#### 2.1.2. Withdrawal/Relapse

Conflicting evidence exists regarding how PPAR-α agonists influence withdrawal from ethanol [28,33]. Bilbao et al. (2016) found that i.p. injection of 5 mg/kg of the endogenous PPAR-α agonist OEA significantly reduced total ethanol withdrawal scores in male rats, and furthermore decreased each of the individual withdrawal signs evaluated (vocalizations, head tremor and rigidity, tail tremor, and body tremor) [28]. Blednov et al. (2016) found that oral administration of 150 mg/kg fenofibrate or 1.5 mg/kg of the dual PPAR-α/γ agonist tesaglitazar actually increased withdrawal severity (handling-induced convulsions score) in male (but not female) mice [33]. The results of these two studies are difficult to compare given the different choices of PPAR-α agonist, dose, and route of administration, withdrawal signs evaluated, and animal models, but do suggest some role of PPAR-α in modulating ethanol withdrawal.

In the same experiments described above, Bilbao et al. (2016) found that both OEA and WY14643 were able to attenuate cue-induced reinstatement of ethanol-seeking after a period of deprivation [28], providing preliminary evidence that PPAR-α agonism may help to prevent alcohol relapse.

Two studies have suggested a role of PPAR-α agonists in reducing nicotine withdrawal signs. Jackson et al. (2017) assessed the impact of PPAR-α agonists on symptoms of precipitated nicotine withdrawal. They observed that WY14643 attenuated anxiety-like behaviors, hyperalgesia, and somatic withdrawal signs, while fenofibrate attenuated only somatic withdrawal signs [40]. Similarly, Donvito et al. (2019) found that exogenous administration of the lipid transmitter OlGly attenuated anxiety-like and somatic signs of nicotine withdrawal [43].

Finally, two studies have provided evidence that PPAR-α agonists can block reinstatement of nicotine-responding following a period of extinction [18,19]. Mascia et al. (2011) found that WY14643 attenuated reinstatement in both rats and monkeys using a procedure that combines both nicotine- and cue-induced reinstatement, and that this effect was reversed by co-administration of the PPAR-α antagonist MK886 [18]. Similarly, Panlilio et al. (2012) found that clofibrate attenuated both nicotine- and cue-induced reinstatement of nicotine responding in monkeys, and that these effects were reversed by pre-treatment with MK866 [19]. The reduction in withdrawal symptoms and the attenuation of both drug- and cue-induced reinstatement suggest that PPAR-α agonists may be useful in preventing relapse in nicotine-dependent smokers.

### 2.2. PPAR-γ Agonists

#### 2.2.1. Consumption/Motivation

Similar to the evidence for PPAR-α agonists, the results of several studies support a role of PPAR-γ agonists in attenuating voluntary consumption and operant self-administration of ethanol [26,29,30,35,37]. In the two-bottle choice paradigm, voluntary ethanol consumption was found to be attenuated by treatment with rosiglitazone [35] and pioglitazone [29,35,37], as well as the dual PPAR-α/γ agonist tesaglitazar [26,29,30]. Stopponi et al. (2011) additionally observed that pioglitazone significantly reduced operant self-administration of ethanol under an FR1 schedule [35]. While one study found that pre-treatment with the PPAR-γ antagonist GW9662 reversed the effects of pioglitazone on voluntary ethanol consumption [35], another study found no effect of GW9662 pre-treatment on the ethanol-reducing effects of the dual PPAR-α/γ agonist tesaglitazar, suggesting that the PPAR-α isoform may be more important in modulating ethanol-related behaviors than the PPAR-γ isoform [30].

Limited evidence suggests that PPAR-γ agonists may not influence ethanol CPP. As described above, Blednov et al. (2016) found no effect of tesaglitazar on ethanol CPP [33].

One study found that pioglitazone reduced operant self-administration of heroin under an FR1 schedule and significantly decreased the breakpoint in a PR schedule [20]. Furthermore, the effects of pioglitazone on self-administration were reversed by pre-treatment with the PPAR-γ antagonist GW9662 [20]. This preliminary evidence suggests that PPAR-γ agonists may be useful in reducing the reinforcing effects of opioids such as heroin.

Two studies have suggested that PPAR-γ agonists can attenuate behavioral sensitization to stimulant drugs [34,41]. Maeda et al. (2007) found that treatment with ciglitazone or pioglitazone during a withdrawal period, but not concurrently with methamphetamine, significantly attenuated behavioral sensitization to methamphetamine, while the PPAR-γ antagonist GW9662 significant augmented behavioral sensitization [34]. Miller et al. (2018) found that treatment with pioglitazone 4 days prior to testing significantly attenuated both the development and expression of behavioral sensitization to cocaine and attenuated lever-pressing for cocaine-associated cues during a period of forced abstinence [41].

#### 2.2.2. Withdrawal/Relapse

Similar to the results for PPAR-α agonists, the current evidence for an effect of PPAR-γ in modulating ethanol withdrawal signs is split [33,35]. As previously described, Blednov et al. (2016) found that the dual PPAR-α/γ agonist tesaglitazar increased withdrawal severity in mice [33]. In contrast, Stopponi et al. (2011) found that oral administration of both 10 and 30 mg/kg pioglitazone significantly reduced total withdrawal signs (composite score of ventromedial distal flexion responses, tail rigidity, and tremors) in rats [35]. While once again significant methodological differences prevent clear comparison of these results, it is important to note that in the same set of experiments, Blednov and colleagues did not find that the effects of tesaglitazar on ethanol-related behaviors were blocked by pre-treatment with the PPAR-γ antagonist GW9662 [30]. Thus, the ability of tesaglitazar to increase ethanol withdrawal severity in their experiment may not have been due to its actions at PPAR-γ.

Two studies have provided evidence for a role of PPAR-γ agonism in blocking reinstatement of ethanol-responding [35,37]. Both studies found that pioglitazone alone significantly attenuated stress-induced reinstatement (using yohimbine as a stressor), but not cue-induced reinstatement [35,37]. However, when pioglitazone was co-administered with naltrexone, there was an attenuation of cue-induced reinstatement [37]. These results suggest that PPAR-γ agonists may be useful in preventing alcohol relapse, possibly to a greater extent when administered concurrently with naltrexone, a non-selective opioid receptor antagonist that is already approved by the United States Food and Drug Administration (FDA) to treat alcohol use disorder [10].

One recent study found that PPAR-γ activation may play a role in nicotine withdrawal. Administration of pioglitazone prior to assessment of nicotine withdrawal attenuated somatic and anxiety-like signs of withdrawal in rats and in wild-type mice with intact PPAR-γ, but not in conditional neuronal PPAR-γ KO mice [42]. In addition, the effect of pioglitazone on both somatic and anxiety-like signs of nicotine withdrawal was blocked by pre-treatment with the PPAR-γ antagonist GW9662 in WT mice [42].

Finally, one study provided evidence that PPAR-γ agonists can reduce opioid withdrawal and relapse [39]. Treatment with pioglitazone significantly attenuated both the development and expression of morphine withdrawal in mice, and the PPAR-γ antagonist GW9662 blocked the ability of pioglitazone to attenuate the expression of morphine withdrawal [39]. Furthermore, pioglitazone significantly attenuated yohimbine- and heroin-induced reinstatement of heroin-responding in rats, while having no effect on cue-induced reinstatement [39]. Previously, the same group reported that pioglitazone significantly attenuated the development of analgesic tolerance to morphine [38], which provides additional evidence for a role of PPAR-γ in the effects of repeated morphine administration.

### 2.3. Summary of Preclinical Evidence

The majority of the preclinical behavioral evidence suggesting a role of PPAR agonists in addiction-like behaviors has focused on ethanol. Currently, the literature strongly supports a role of PPAR-α agonists (gemfibrozil, fenofibrate, OEA, and WY14643), and PPAR-γ agonists (rosiglitazone and pioglitazone) or a dual PPAR-α/γ agonist (tesaglitazar) to a lesser extent, in attenuating the voluntary consumption and reinforcing properties of ethanol in rodents. Limited evidence suggests that the PPAR-α agonist fenofibrate may additionally reduce the rewarding properties of ethanol, as assessed in the CPP paradigm. While agonists at both PPAR-α (OEA and fenofibrate) and PPAR-γ (pioglitazone) seem to have some role in modulating ethanol withdrawal signs, the nature of this role is unclear. However, the evidence does suggest that PPAR agonists may be useful in reducing the likelihood of alcohol relapse after a period of abstinence. PPAR-α agonists (OEA and WY14643) were shown to attenuate cue-induced reinstatement of ethanol-seeking, while a PPAR-γ agonist (pioglitazone) was shown to attenuate stress-induced reinstatement (and possibly also cue-induced reinstatement when co-administered with naltrexone).

Robust evidence from a limited number of studies strongly supports a role of PPAR-α (and possibly PPAR-γ) agonists in modulating nicotine-related behaviors in both rodents and non-human primates. The PPAR-α agonists methyl-OEA, WY14643, and clofibrate were found to reduce the reinforcing properties of nicotine. In addition, WY14643, fenofibrate, and OlGly were found to reduce the rewarding effects of nicotine in the CPP paradigm. WY14643 was shown to decrease behavioral and somatic signs of nicotine withdrawal, while both WY14643 and clofibrate reduced drug- and cue-induced reinstatement of nicotine-seeking. Finally, the PPAR-γ agonist pioglitazone reduced somatic and anxiety-like signs of nicotine withdrawal.

Preliminary evidence suggests that PPAR-γ agonists may have a role in modulating opioid-related behaviors. Studies found that pioglitazone was able to reduce the reinforcing effects of heroin in an operant self-administration paradigm, decrease both drug- and stress-induced reinstatement of heroin-seeking, and reduce the development and expression of morphine tolerance and withdrawal.

Finally, there seems to be a role of PPAR agonists in psychostimulant-related behaviors, yet the evidence is mixed. The PPAR-γ agonists ciglitazone and pioglitazone attenuated behavioral sensitization to methamphetamine, while pioglitazone attenuated behavioral sensitization to cocaine. Additionally, the endogenous PPAR-α agonist OEA attenuated behavioral sensitization to cocaine and cocaine CPP, but through a PPAR-α-independent mechanism. However, it is important to note that studies of nicotine-related outcomes found no effect of PPAR-α agonists on operant self-administration of cocaine or cocaine CPP.

## 3. Clinical or Human Laboratory Evidence

A summary of the methodological details and relevant findings of the human studies reviewed is provided in Table 2.

### 3.1. PPAR-α Agonists

Two published placebo-controlled studies have evaluated the potential of PPAR-α agonists in treatment of nicotine dependence [44,45]. Perkins et al. (2016) recruited nicotine-dependent smokers high in quit interest for a double-blind, counterbalanced, crossover trial with a target dose of 160 mg of fenofibrate administered once daily for 4 days following a 4-day dose run-up period [44]. There was no difference between fenofibrate and placebo on quit days, the primary outcome of the trial. In addition, there were no drug effects on any of the secondary outcomes, including pre-quit smoking reinforcement (i.e., number of puffs taken from participants’ preferred brand of cigarettes and self-reported rewarding effects), craving responses during a cue reactivity task, and mean daily reductions in smoking [44]. In support of these negative findings, our lab found no effect of gemfibrozil (600 mg administered orally twice daily) on total self-reported days abstinent in a sample of nicotine-dependent smokers intent on quitting [45]. Similarly, we found no effects on secondary outcomes including a forced choice procedure (i.e., reinforcing effects) and both physiological and subjective measures of cue reactivity. In sum, despite the compelling preclinical evidence, the limited human evidence has not supported a role of PPAR-α agonists in treating nicotine dependence.

### 3.2. PPAR-γ Agonists

Three placebo-controlled studies have examined the potential for PPAR-γ agonists in modulating opioid-related outcomes [46,47,48]. In a sample of healthy, non-medical users of prescription opioids, there was no effect of 15 or 45 mg oral pioglitazone administered daily for 2–3 weeks on self-reported positive and negative subjective effects of oxycodone in a single-blind, within-subjects design [46]. In addition, pioglitazone had no impact on self-reported drug wanting (opioids, alcohol, cannabis, and tobacco) during the maintenance phases [46]. In a follow-up study, Jones and colleagues assessed the effects of 45 mg oral pioglitazone administered daily for 3 weeks in a sample of non-treatment-seeking adults with an opioid use disorder using a single-blind, randomized, between-subjects design [47]. Pioglitazone did not alter the reinforcing effects of heroin, its abuse liability, or cue reactivity, though self-reported ratings of “I want heroin” were significantly reduced [47]. Finally, Schroeder et al. (2018) assessed the potential for pioglitazone as an adjunct pharmacotherapy for patients with an opioid use disorder undergoing buprenorphine taper [48]. Pioglitazone treatment had no effect on withdrawal severity, and may actually have increased subjective withdrawal; yet, this trial was limited by very low recruitment numbers [48].

Two additional studies have investigated the role of pioglitazone in nicotine dependence and cocaine use disorder. In a single-blind, between-subjects laboratory study of nicotine-dependent smokers not interested in quitting, compared to placebo treatment, 45 mg oral pioglitazone administered daily for 3 weeks decreased self-reported measures of nicotine craving, though had minimal or no impact on reinforcing effects, self-reported positive or negative subjective effects, or cue reactivity [49]. In a pilot study to assess the potential of pioglitazone to target craving and white matter integrity in treatment-seeking adults with cocaine use disorder, daily administration of 45 mg oral pioglitazone for 12 weeks conferred benefit over placebo in reducing cocaine craving [50].

Taken together, the limited available human evidence suggests that the PPAR-γ agonist pioglitazone may be beneficial in reducing heroin, nicotine, and cocaine craving. However, it remains unclear how PPAR-γ agonists may impact more direct measures of treatment efficacy such as quit days or reductions in drug use.

## 4. Synthesis of the Preclinical and Human Evidence

Given the robust preclinical evidence that both PPAR-α and PPAR-γ play a role in addiction-related behaviors, the lack of significant findings from human studies is somewhat surprising. For example, multiple preclinical studies demonstrated that PPAR-α agonists were effective in reducing the reinforcing and rewarding properties of nicotine and reducing nicotine withdrawal and reinstatement of nicotine-seeking [18,19,40,43], yet two human trials found no effect of the PPAR-α agonists fenofibrate [44] or gemfibrozil [45] on smoking cessation outcomes. Potential explanations for the poor concordance between the animal and human evidence to data are discussed below.

Perhaps the most salient discordance between the animal and human literature is the complete lack of placebo-controlled trials of PPAR agonists for treatment of alcohol use disorder. One Phase II trial of pioglitazone for treatment of alcohol craving and other alcohol-related outcomes in adults with alcohol use disorder (ClinicalTrials.gov identifier: NCT01631630) was terminated due to feasibility problems. A similar Phase II trial of fenofibrate (ClinicalTrials.gov identifier: NCT02158273) has been completed, though the results are unpublished. The most consistently reported and robust addiction-related outcome associated with PPAR agonists in the preclinical literature is a reduction in voluntary consumption of ethanol. Yet, as of this writing, the potential for PPAR agonists in treatment of alcohol use disorders in human has not been reported in the published literature. Thus, this is an important priority for future research. Currently, most pharmacotherapies available for the treatment of substance use disorders are substance-specific (although some are able to affect different substance use disorders). Therefore, it would be important to study the role of PPAR agonists in various substances use disorders, as it is unlikely that a single drug would be able to cure all substance use disorders.

The choice of PPAR agonist and dose is likely an important source of the poor translation from animal to human studies. For example, Jones and colleagues noted that the pioglitazone dosing parameters they employed were based on clinical utility in treating type-II diabetes [46,49], which may not be sufficient to elicit an effect in attenuating the abuse liability or reinforcing effects of opioids or nicotine. Similarly, while the preclinical evidence for a role of fibrate drugs in attenuating nicotine-related behaviors came from a study administering clofibrate [19], Perkins et al. (2016) used fenofibrate instead, as clofibrate was removed from the U.S. market due to its adverse effects [44]. Fibrate drugs, in general, may be less effective in reducing the rewarding and reinforcing effects of nicotine compared to experimental compounds such as WY14643 [40]. This could be due to the poor blood-brain barrier penetrance of fibrates like fenofibrate [51,52] or the low potency and PPAR-α selectivity of fenofibrate [53]. It should be noted in general that the PPAR agonists available do not act with 100% selectivity on specific PPAR isoforms and therefore, action on multiple PPAR isoforms is a possibility that should be kept in mind while interpreting the research results. Thus, different dosing paradigms, or perhaps more potent and selective PPAR agonists, may be needed to elicit clinically meaningful outcomes.

Similarly, species differences in the distribution and signaling of PPARs could also play in a role in the negative human findings. For example, significant differences in the expression [54] and activity [55] of hepatic PPAR-α has been demonstrated in human and rat, in part due to differences in the PPREs of target genes [55]. In addition, species differences have been demonstrated in PPAR-α binding of and response to specific ligands (including clofibrate) [56]. While one recent study did suggest a similar brain distribution of PPARs in adult mice and humans [57], it is still possible that species differences in PPAR-ligand dynamics and in PPAR distribution and signaling could limit the translation of findings from animal models to human studies. The fact that there is poor inter-species comparability in the activity of PPAR agonists is not something unique for PPAR ligands. There have been multiple cases of drugs that appear to be effective in preclinical studies that have not been effective in clinical trials. For example, despite an extensive preclinical literature showing that corticotropin-releasing hormone (CRH) acting via its CRH1 receptor can affect alcohol-seeking behavior, the drug pexacerfont, a CRH1 brain-penetrant antagonist, had no clinical efficacy in a clinical trial in subjects with alcohol dependence [58]. Although it is yet too early to determine if PPAR agonists would similarly fail in humans, this remains a possibility.

Another possibility is simply that the published human studies were underpowered and too few in number to draw conclusions. Jones and colleagues note in two of their pioglitazone studies that they did not reach their recruitment goals [47,49]. Schroeder et al. (2018) noted significant difficulty in recruiting for their study of pioglitazone effects on opioid withdrawal during buprenorphine taper, reaching less than half of their target recruitment [48]. Schmitz et al. (2017), despite finding a potentially meaningful effect of pioglitazone on cocaine craving, note that their study was a pilot trial not specifically powered to detect a difference between drug conditions [50]. Appropriately powered randomized clinical trials are required to clarify the human evidence.

Finally, one possibility that has yet to be considered is the role of sex-related factors in the behavioral pharmacology of PPAR agonists. As seen in Table 1, the overwhelming majority of preclinical studies reviewed included only male animals in their experiments. In the two papers that did report sex differences, the PPAR-α agonist fenofibrate was shown to have more consistent and robust effects on ethanol-related outcomes (voluntary consumption and withdrawal severity) in male mice compared to female mice [30,33]. Furthermore, emerging evidence has found higher expression of PPAR-α mRNA and protein in immune cells of male rodents [59,60]; a role of PPAR-α in neuroprotection [61] and hippocampal synaptic plasticity [62] in male, but not female, rodents; and sex differences in the adverse effects and pharmacokinetics of PPAR-γ agonists such as pioglitazone in humans [63]. Given that all human studies reviewed included female participants (though consistently a small minority), sex differences in the effects of PPAR agonists on drug-related outcomes could have obscured overall drug effects.

## 5. Future Directions

Given the robust preclinical evidence for an effect of PPAR-α agonists in particular on ethanol-related outcomes, an important first step in moving forward with translating the animal evidence will be conducting human laboratory studies to determine if PPAR agonists (such as gemfibrozil or fenofibrate) modulate the subjective and reinforcing effects of alcohol. Subsequent to this, or in parallel, pilot RCTs to evaluate the efficacy and feasibility of administering PPAR agonists in alcohol use disorder will be necessary.

PPAR-α agonists showed promise for targeting nicotine-related behaviors in animal models, yet two adequately powered human trials found no benefit of fenofibrate or gemfibrozil on smoking cessation or other nicotine-related outcomes. It is possible that these agonists do not have sufficient pharmacological activity at PPAR-α to elicit clinically meaningful outcomes. Indeed, preclinical evidence has shown that more potent compounds like WY14643 confer benefit in attenuating nicotine-related behaviors over fibrates [40]. Selective PPAR modulators (SPPARMS), such as the highly potent and selective PPAR-α agonist K-877, have already shown some promise in treating dyslipidemias and insulin resistance with favorable adverse effect profiles compared to approved drugs such as fenofibrate [53]. If these compounds continue to show efficacy with limited adverse effects, it may be worth testing SPPARMS as smoking cessation drugs in RCTs.

It is possible that targeting PPAR isoforms alone may not be sufficient to treat addictions. For example, as discussed previously, pioglitazone was more effective in reducing reinstatement to ethanol-seeking when it was co-administered with naltrexone [37], an opioid receptor antagonist, suggesting some degree of synergy between PPAR activation and opioid receptor inhibition. Similarly, it has been proposed that simultaneous inhibition of FAAH and activation of PPARs may have an additive or even synergistic effect in treating cancers [64], and this approach may similarly hold promise in the context of addiction pharmacotherapy [65]. Future studies should consider possible synergistic effects that could be achieved by modulation of multiple signaling systems.

It will also be important to validate that the PPAR ligands that are used for SUD treatment are able to occupy/activate brain PPARs. Use of brain imaging approaches such as positron emission tomography could be useful for such target engagement validation. This is critical as some of the previous drug indications for PPAR ligands were likely mediated through PPAR action at the periphery [66].

The PPAR-β/δ isoform was not discussed in this review due to the lack of evidence implicating PPAR-β/δ agonists in addiction-related behaviors. However, it is important to note that PPAR-β/δ is present in the rodent brain at higher levels than the other two isoforms [67] and may play a role in regulating the expression and activity of PPAR-α and PPAR-γ [68]. Furthermore, limited evidence has suggested a role of PPAR-β/δ in neurodevelopmental and neurodegenerative disorders, possibly related to its anti-inflammatory properties [13]. Thus, future studies should investigate the role of PPAR-β/δ agonists in behavioral models of addiction.

A robust body of literature has demonstrated sex-related variability in the effects of common drugs of abuse and in addiction-related processes across animal and human studies [69,70,71], and emerging evidence suggests similar sex-related variability in the pharmacology of PPAR ligands and in PPAR signaling and function [59,61,62,63]. Considering sex as a biological variable in future animal studies of PPAR agonists and addiction-related behaviors will be another important next step.

Taken together, this review highlights the robust findings obtained in preclinical studies with agonists at both the PPAR-α and PPAR-γ isoforms that appear effective to reduce both positive and negative reinforcing properties of various drugs of abuse. However, the clinical findings are so far mixed and seem to indicate that the potential is much lower in human subjects. At this point, it is still important to perform small-scale appropriately powered proof of principle studies with PPAR drugs engaging brain PPARs to validate these findings in humans. Positive signals should then be followed by larger RCTs for further validation.

## Figures and Tables

**Table 1:** Overview of methodological details and primary findings of the key studies providing behavioral evidence for a role of PPAR agonists in modulating addiction-related behaviors in animal models.

Reference	Species/Strain and Sex	Addiction Model and Task	PPAR Agonist, Dose, and Route of Administration	Treatment Regimen	Primary Findings
Maeda et al., 2007 [34]	Male ICR mice	Behavioral sensitization to methamphetamine	0.5–5 μg i.c.v. CIG and PIO (PPAR-γ)	Once daily administration either for 5 days concurrently with methamphetamine or for 6 days during the withdrawal period	No effect of CIG or PIO (5 μg) when administered concurrently with methamphetamine When administered during the withdrawal period, both CIG and PIO (at 5 μg, but not 0.5 μg or 1.5 μg) attenuated behavioral sensitization, while 1.5 and 5 μg (but not 0.5 μg) GW9662 (PPAR-γ antagonist) augmented behavioral sensitization
Barson et al., 2009 [25]	Male Sprague-Dawley rats	Voluntary ethanol consumption (2BC paradigm)	50 mg/kg p.o. GEM (PPAR-α)	One gavage 2 h prior to 4-h access to ethanol	GEM reduced intake of 7% ethanol, with a significant effect at 1 h and 4 h (and reduced ethanol consumption during the first hour of access in a separate experiment)
Mascia et al., 2011 [18]	Male Sprague-Dawley rats Male squirrel monkeys	Operant SA (FR5 schedule of i.v. nicotine) (rats) Nicotine seeking and relapse (nicotine/cue-induced reinstatement) (rats) Nicotine discrimination (rats) Operant SA (FR10 schedule of i.v. nicotine or cocaine) (monkeys) Nicotine seeking and relapse (nicotine/cue-induced reinstatement) (monkeys)	20 or 40 mg/kg i.p. WY14643 and 10 mg/kg i.p. methOEA (PPAR-α) (rats) 10, 20, or 40 mg/kg i.m. WY14643 and 10 mg/kg i.m. methOEA (PPAR-α) (monkeys)	Single injections of WY14643 20 min prior or methOEA 40 min prior to SA sessions (rats and monkeys) WY14643 20 min prior to reinstatement (rats and monkeys) WY14643 substituted for training dose of nicotine and co-administered with various doses of nicotine during discrimination sessions (rats)	Both WY14643 and methOEA (at all tested doses) reduced nicotine SA in rats and monkeys; co-administration with MK886 (PPAR-α antagonist) attenuated this effect in monkeys WY14643 attenuated nicotine/cue-induced reinstatement at both doses tested in rats and monkeys; MK886 attenuated this effect in monkeys WY14643 had no effect on cocaine SA in monkeys or nicotine discrimination in rats
Stopponi et al., 2011 [35]	Male msP (alcohol-preferring Marchigian Sardinian) rats	Voluntary ethanol consumption (2BC paradigm) Operant SA (FR1 schedule of oral ethanol) Ethanol seeking and relapse (stress- and cue-induced reinstatement) Ethanol withdrawal (ventromedial distal flexion response, tail stiffness/rigidity, and tremors)	10 or 30 mg/kg p.o PIO or ROSI (PPAR-γ)	Twice daily treatment (12 h and 1 h prior to dark period) for 7 consecutive days (2BC) or 3 consecutive days (2BC with antagonism treatment) Twice daily treatment every fourth day (SA)Single treatment 12 h and 1 h prior to reinstatement test and evaluation of withdrawal symptoms	PIO significantly reduced voluntary intake of 10% ethanol on all treatment days at 30 mg/kg, but only on treatment days 5 and 7 at 10 mg/kg; ROSI also significantly reduced intake at the 30 mg/kg dose on all treatment days except day 4, while only on days 1, 2, and 7 at the 10 mg/kg dose The effect of PIO (30 mg/kg) on ethanol intake was attenuated by pre-treatment with 5 µg GW9662 (PPAR-γ antagonist) across all 3 treatment days PIO (at 30 mg/kg, but not 10 mg/kg) significantly reduced operant SA of 10% ethanol Pre-treatment with both doses of PIO significantly attenuated yohimbine-induced reinstatement of ethanol-seeking, but had no effect on cue-induced reinstatement PIO (at both doses) significantly reduced total withdrawal signs
Panlilio et al., 2012 [19]	Male Sprague-Dawley rats Male squirrel monkeys	Operant SA (FR1 or FR5 schedule of i.v. nicotine) (rats) Nicotine discrimination (rats) Operant SA (FR10 schedule of i.v. nicotine) (monkeys) Nicotine seeking and relapse (nicotine- and cue-induced reinstatement) (monkeys)	100, 200, or 300 mg/kg i.p. CLO (PPAR-α) (rats) 25, 50, or 100 mg/kg i.m. CLO (PPAR-α) (monkeys)	Single injections once daily beginning two days prior to 18 days of testing (FR1, rats) Single injections once daily for 3 days (FR5, rats; FR10, monkeys) Single injection prior to priming injection of nicotine (reinstatement, monkeys) Single injection 100 min prior to discrimination sessions (rats)	CLO (300 mg/kg) prevented the acquisition of nicotine SA in naïve rats CLO decreased SA of nicotine in experienced rats (at all three doses) and monkeys (at 50 mg/kg and 100 mg/kg, but not 25 mg/kg); this effect was attenuated by pre-treatment with 3 mg/kg MK886 (PPAR-α antagonist) In monkeys, 100 mg/kg CLO attenuated both nicotine- and cue-induced reinstatement of nicotine-seeking; these effects were attenuated with MK886 pre-treatment CLO did not alter nicotine discrimination in rats
Bilbao et al., 2013 [36]	Male PPAR-α KO mice and WT counterparts	Behavioral sensitization to cocaine Cocaine CPP	1, 5, or 20 mg/kg i.p OEA (PPAR-α)	Single injection prior to tests (motor response and CPP) followed by injections every other day for 3 additional days (sensitization)	OEA (5 mg/kg and 20 mg/kg, but not 1 mg/kg) attenuated acute cocaine-induced motor activation and sensitization to the motor effects of cocaine OEA attenuated cocaine CPP at 1 and 5 mg/kg and completely abolished the development of CPP at 20 mg/kg The ability of OEA (20 mg/kg) to attenuate cocaine sensitization and CPP was intact in PPAR-α KO mice
Stopponi et al., 2013 [37]	Male msP rats	Voluntary ethanol consumption (2BC paradigm) Ethanol seeking and relapse (stress- and cue-induced reinstatement)	10 or 30 mg/kg p.o. PIO (PPAR-γ)	Two treatments (12 h and 1 h prior to dark period) prior to testing sessions	PIO (30 mg/kg, but not 10 mg/kg) reduced intake of 10% ethanol at 24 h (but not 2 or 8 h); 10 mg/kg PIO co-administered with 0.25 mg/kg naltrexone also significantly reduced intake at 8 and 24 h PIO (at both doses) and co-administration of 1 mg/kg naltrexone with either dose of PIO significantly attenuated yohimbine-induced reinstatement of ethanol-seeking PIO alone did not significantly alter cue-induced reinstatement of ethanol-seeking, but co-administration of 1 mg/kg naltrexone with either PIO dose did
De Guglielmo et al., 2014 [38]	Male C57 mice and conditional neuronal PPAR-γ KO mice and WT counterparts	Analgesic tolerance to morphine	10 or 30 mg/kg p.o. PIO (PPAR-γ)	Single gavage prior to morphine injections for 9 days (or only on days 8 and 9 for reversal of morphine tolerance experiments)	PIO (at both doses) attenuated the development of tolerance to the analgesic effects of morphine; this effect was blocked by pretreatment with 5 mg/kg GW9962 (PPAR-γ antagonist) and was absent in the PPAR-γ KO mice compared to their WT counterparts GW9962 alone accelerated the development of morphine tolerance PIO (at both doses) also reversed morphine tolerance when administered only on the last two days of treatment
Ferguson et al., 2014 [26]	Male C57BL/6J mice	Voluntary ethanol consumption (2BC paradigm)	150 mg/kg p.o. FEN (PPAR-α) 75 mg/kg p.o. BEZA (pan-PPAR) 1.5 mg/kg p.o. TESA (dual PPAR-α/γ)	Single treatment for 8 days (ethanol consumption measured on days 5 and 6)	FEN and TESA decreased voluntary consumption of and preference for 15% ethanol, while BEZA had no significant effect
Karahanian et al., 2014 [27]	Male UChB (selectively bred high-drinker) rats	Voluntary ethanol consumption (24-h 2BC and limited 2BC drinking in the dark paradigms)	50 mg/kg p.o. FEN (PPAR-α)	Single daily treatment for 14 consecutive days following 60 days of continuous free choice of ethanol or water	In the 24-h access paradigm, FEN reduced voluntary consumption of 10% ethanol, starting on day 4 of treatment and reaching a maximum reduction at day 12 In the drinking in the dark paradigm, FEN significantly reduced ethanol intake, starting on day 2 and reaching a maximum reduction at day 5
Blednov et al., 2015 [29]	Male C57BL/6J mice	Voluntary ethanol consumption (24-h 2BC and limited 2BC drinking in the dark paradigms)	10 or 30 mg/kg p.o. PIO (PPAR-γ) 50 or 150 mg/kg p.o. FEN (PPAR-α) 10 mg/kg p.o. GW0742 (PPAR-δ/β) 1.5 mg/kg p.o. TESA (dual PPAR-α/γ) 25 or 75 mg/kg p.o. BEZA (pan PPAR-α/γ/δ/β)	Once daily treatment for up to 10 days following 2 days of saline treatment	In the 24-h access paradigm, PIO (30 mg/kg), FEN (150 mg/kg), and TESA reduced intake of and preference for 15% ethanol; BEZA (75 mg/kg) reduced preference, but not intake; GW0742 had no effect In the drinking in the dark paradigm, FEN (150 mg/kg), TESA (1.5 mg/kg), and BEZA (75 mg/kg) reduced intake and preference; PIO and GW0742 had no effect
De Guglielmo et al., 2015 [20]	Male Wistar rats	Operant SA (FR1 or PR schedule of i.v. heroin)	30 or 60 mg/kg p.o. PIO (PPAR-γ)	Twice-daily treatment (12 and 1 h prior to SA session) for 5 days	PIO significantly reduced heroin SA under an FR1 schedule (at 60 mg/kg, but not 30 mg/kg) and significantly decreased the breakpoint in the PR schedule (at 30 and 60 mg/kg); the reduction in responding under FR1 with 60 mg/kg PIO was blocked by pre-treatment with 5 mg/kg GW9662 (PPAR-γ antagonist)
Bilbao et al., 2016 [28]	Male Wistar rats	Voluntary ethanol consumption (2BC paradigm) Operant SA (FR1 schedule of oral ethanol) Ethanol seeking and relapse (cue-induced reinstatement) Ethanol withdrawal (vocalizations, head tremor and rigidity, tail tremor, and body tremor)	1, 5, or 20 mg/kg i.p OEA (PPAR-α) 5, 20, or 40 mg/kg i.p. WY14643 (PPAR-α)	Single injections 30 min prior to testing sessions	OEA (5 mg/kg) significantly decreased voluntary intake of 10% ethanol at all time points (2, 4, and 6 h); this effect was reversed by pre-treatment with 1 mg/kg GW6471 (PPAR-α antagonist) OEA (5 mg/kg and 20 mg/kg, but not 1 mg/kg) and WY14643 (20 mg/kg and 40 mg/kg, but not 5 mg/kg) significantly decreased SA of 10% ethanol OEA (5 mg/kg and 20 mg/kg, but not 1 mg/kg) and WY14643 (20 and 40 mg/kg) significantly attenuated cue-induced reinstatement of ethanol-seeking OEA (5 mg/kg, but not 1 mg/kg) and WY14643 (20 mg/kg) decreased ethanol SA following a deprivation periodOEA (5 mg/kg) significantly reduced ethanol withdrawal scores
Blednov et al., 2016 [30]	Male and female C57BL/6J and PPAR-α KO mice	Voluntary ethanol consumption (continuous and intermittent 2BC paradigm)	10, 50, 100, or 150 mg/kg p.o. FEN (PPAR-α) 1.5 mg/kg p.o. TESA (dual PPAR-α/γ)	Once daily treatment for up to 14 days after 2 days of saline treatment	In the continuous access paradigm, FEN reduced both intake of and preference for 15% ethanol (at 100 and 150 mg/kg, but not 10 mg/kg or 50 mg/kg) in male, but not female, mice; TESA reduced both intake and preference in both male and female mice In the intermittent (every other day) access paradigm, FEN (150 mg/kg, but not 100 mg/kg) reduced both intake and preference in male and female mice Pre-treatment with 5 mg/kg MK886 (PPAR-α antagonist), but not 5 mg/kg GW9662 (PPAR-γ antagonist), reduced the effect of FEN on ethanol intake; pre-treatment with GW9662 or MK886 did not block the effects of TESA on ethanol intake Both FEN and TESA had no effect on ethanol consumption in mice lacking PPAR-α
Blednov et al., 2016 [33]	Male and female C57BL/6J and B6 × 129S4 mice	Ethanol CPPEthanol withdrawal (handling-induced convulsions)	150 mg/kg p.o. FEN (PPAR-α) 1.5 mg/kg p.o. TESA (dual PPAR-α/γ)	Once daily treatment for the duration of each experiment after 2 days of saline treatment	No effect of either agonist on CPP in male B6x129S4 mice FEN increased withdrawal severity in male mice of both genotypes, while TESA increased withdrawal severity in only the B6x129S4 male mice; neither drug significantly altered withdrawal in female mice
De Guglielmo et al., 2017 [39]	Male Wistar rats and male CD1 mice	Morphine withdrawal (jumps, paw tremors, teeth chattering, and wet dog shakes) Heroin seeking and relapse (stress-, cue-, and heroin-induced reinstatement)	10, 30, or 60 mg/kg p.o. PIO (PPAR-γ)	Single treatment 1 h prior to morphine injection the evening of day 5 and morning of day 6 (withdrawal expression) Treatment twice daily (12 h and 1 h prior to tests) for 5 consecutive days, then again on the morning of day 6 1 h prior to final morphine injection (withdrawal development) Two treatments, 12 h and 1 h prior to reinstatement tests	In mice, PIO (10 and 30 mg/kg) attenuated the expression of morphine withdrawal and the development of morphine withdrawal (at 30 mg/kg); pre-treatment with 5 mg/kg GW9662 (PPAR-γ antagonist) reversed the effect of PIO on expression of withdrawal In rats, PIO significantly reduced yohimbine-induced reinstatement (at 30 mg/kg, but not 10 mg/kg) and heroin-induced reinstatement (at 30 mg/kg and 60 mg/kg, but not 10 mg/kg) of heroin-seeking, but had no effect on cue-induced reinstatement (at 10, 30, or 60 mg/kg)
Haile & Kosten, 2017 [31]	Wistar rats (sex not reported)	Operant SA of ethanol (FR2 and PR)	25, 50, or 100 mg/kg p.o. FEN (PPAR-α)	Single treatment 1 h prior to test sessions for 5 consecutive days (four days of FR2 schedule then one day of PR schedule)	Under the FR2 schedule, there was a significant difference between all doses tested, though the effect was dependent on day (by day 4, all three active doses of FEN significantly decreased active lever presses for 10% ethanol) Under the PR schedule, all three doses of FEN reduced active lever presses
Jackson et al., 2017 [40]	Male ICR mice	Nicotine (and cocaine) CPP Nicotine withdrawal (anxiety-like behavior, somatic withdrawal signs, and hyperalgesia)	0.3, 0.6, 1, and 5 mg/kg i.p. WY14643 (PPAR-α) 1, 9, 50, or 100 mg/kg i.p. FEN (PPAR-α)	For CPP experiments, WY14643 was administered 15 min prior to and FEN 1 h prior to nicotine Following 14 days of infusion with nicotine, mice were given a single treatment with WY14643 15 min prior to or FEN 1 h prior to precipitated withdrawal on day 15	WY14643 (at all three doses) significantly attenuated nicotine CPP WY14643 did not shift the potency of nicotine in the CPP paradigm WY14643 (1 mg/kg) did not attenuate cocaine CPP FEN attenuated nicotine CPP at 50 mg/kg (not 1, 9, or 100 mg/kg) WY14643 attenuated signs of nicotine withdrawal (anxiety-like behaviors and hyperalgesia attenuated at 5 mg/kg only; somatic withdrawal symptoms attenuated at 1 and 5 mg/kg; no effect of 0.3 mg/kg) FEN did not attenuate anxiety-like behaviors or hyperalgesia at either dose tested (50 or 100 mg/kg), but did attenuate somatic withdrawal symptoms at 100 mg/kg
Rivera-Meza et al., 2017 [32]	Male UChB rats	Voluntary ethanol consumption (2BC paradigm) Ethanol CPP	25, 50, or 100 mg/kg p.o. FEN (PPAR-α)	Following 60 days free choice between ethanol and water, rats were treated once daily for 14 days (in the CPP experiment, ethanol access was restricted during this period, and testing occurred on day 14 of FEN treatment) In a separate experiment, rats were deprived of ethanol on day 60 and treated once during two deprivation periods (days 61–74 and 103–116), voluntary consumption of ethanol was once again measured after each of these two periods	FEN (all three doses) significantly decreased voluntary consumption of 10% ethanol beginning on day 2 of treatment and continuing for the duration of treatment FEN (50 mg/kg) prevented the development of ethanol CPP FEN (50 mg/kg) significantly decreased voluntary consumption of ethanol following both periods of deprivation
Miller et al., 2018 [41]	Male Sprague-Dawley rats	Behavioral sensitization to cocaine Cocaine cue reactivity (lever-pressing for cocaine-associated cues during forced abstinence)	50 mg of PIO per kg of chow	PIO treatment initiated 4 days prior to behavioral sensitization protocol and immediately following final session of cocaine SA (continued during 30-day forced abstinence period)	PIO reduced both the development and expression of behavioral sensitization to cocaine PIO reduced cue reactivity following prolonged abstinence from cocaine; this effect was attenuated by pre-treatment with 1 mg/kg GW9662 (PPAR-γ antagonist)
Domi et al., 2019 [42]	Male Wistar rats and conditional neuronal PPAR-γ KO mice and WT counterparts	Nicotine withdrawal (somatic withdrawal signs and anxiety-like behaviors)	15 or 30 mg/kg p.o. PIO (PPAR-γ)	Two treatments, 12 h and 1 h prior to assessment of withdrawal	PIO (at both doses) reduced somatic signs of nicotine withdrawal and anxiety-like behaviors in rats and WT mice, but had no effect in conditional neuronal PPAR-γ KO mice; the effect of 30 mg/kg PIO on somatic and anxiety-like withdrawal signs was blocked by pre-treatment with GW9662 (PPAR-γ antagonist) in WT mice
Donvito et al., 2019 [43]	Male ICR mice	Nicotine withdrawal (anxiety-like behavior and somatic withdrawal signs) Nicotine (and morphine) CPP	10, 30, or 60 mg/kg i.p. OlGly (PPAR-α)	Single injection 15 min prior to nicotine injection in the CPP experiments or to precipitated withdrawal	OlGly (at 60 mg/kg, but not 10 mg/kg or 30 mg/kg) significantly attenuated anxiety-like and somatic nicotine withdrawal signs OlGly (at all three doses) attenuated the development of nicotine (but not morphine) CPP; this effect was blocked by pre-treatment with 2 mg/kg GW6471 (PPAR-α antagonist)

2BC, two-bottle choice; BEZA, bezafibrate; CIG, ciglitazone; CLO, clofibrate; CPP, conditioned place preference; FEN, fenofibrate; FR, fixed ratio; GEM, gemfibrozil; i.c.v., intracerebroventricular; i.m., intramuscular; i.p., intraperitoneal; i.v., intravenous; KO, knock-out; methOEA, methyl oleoylethanolamide; OEA, oleoylethanolamide; OlGly, N-Oleoyl-glycine; PIO, pioglitazone; p.o., per os (oral); ROSI, rosiglitazone; SA, self-administration; TESA, tesaglitazar; WT, wild-type.

**Table 2:** Overview of methodological details and primary findings of the key clinical and human laboratory studies of PPAR agonists in drug-related outcomes.

References	Study Sample	PPAR Agonist, Dose, and Route of Administration	Study Design	Primary Findings
Jones et al., 2016 [46]	Healthy non-medical users of prescription opioids, N = 17 (15 M, 2 F), 21–55 years old (mean 35 years)	15 or 45 mg p.o. PIO (PPAR-γ)	Single-blind, within-subjects, placebo-controlled design. Participants received PIO doses in ascending order and maintained on each dose for 2–3 weeks. Subjective, analgesic, and physiological effects of oral oxycodone examined at the end of each maintenance phase.	No effect of PIO on self-reported positive or negative subjective effects of oxycodone In addition, PIO did not affect drug wanting (opioids, alcohol, cannabis, or tobacco) during the maintenance phase
Perkins et al., 2016 [44]	Nicotine-dependent smokers high in quit interest, N = 38 (27 M, 11 F), 18–5 years old (mean 30.3 years)	160 mg p.o. FEN (PPAR-α)	Double-blind, within-subjects, counterbalanced, placebo-controlled design. Participants received FEN for 8 days (4-day dose run-up followed by 4-day quit period). A week of ad libitum smoking separated the two quit periods. Self-report of no smoking and expired-air CO < 5 ppm were assessed daily during quit periods. Secondary outcome measures included acute smoking reinforcement and cue reactivity (pre-quit) and amount of daily smoking exposure (post-quit).	FEN did not increase quit days compared to placebo Additionally, FEN had no impact on acute smoking reinforcement (SA paradigm), cue-induced craving, or mean daily smoking
Jones et al., 2017 [49]	Nicotine-dependent smokers not interested in quitting, N = 27 (14 active, 13 placebo; 25 M, 2 F), 21–55 years old (mean 44.9 years in active group, 41.6 years in placebo group)	45 mg p.o. PIO (PPAR-γ)	Single-blind, between-subjects, randomized, placebo-controlled design. Participants received PIO daily for 3 weeks. Laboratory testing (reinforcing effects, cue reactivity, subjective effects, and physiological effects) began after the first week of nicotine patch stabilization.	PIO did not alter the reinforcing effects of nicotine (verbal choice and progressive choice paradigms) or subjective/physiological reactivity to smoking cues PIO had minimal impact on positive subjective effects (increased one measure of nicotine “high”) and no impact on negative subjective effects PIO decreased subjective ratings of “craving” and “desire”
Schmitz et al., 2017 [50]	Treatment-seeking adults with cocaine use disorder, N = 30 (15 active, 15 placebo; 22 M, 8 F), 18–60 years old (mean 48.3 in active group, 47.4 in placebo group)	Target dose of 45 mg p.o. PIO (PPAR-γ)	Double-blind, between-subjects, randomized placebo-controlled pilot trial design. Following a 1-week baseline period and a 2-week dose titration period, participants were maintained on 45 mg/day PIO for duration of study (12 weeks total). Periodic measures of craving and cocaine use.	High probability that PIO conferred benefit over placebo in reducing cocaine craving In addition, there was evidence that PIO decreased the odds of using cocaine during the treatment period
Gendy et al., 2018 [45]	Nicotine-dependent smokers high in quit interest, N = 27 (17 M, 10 F), 19–65 years old (mean 43 years old)	2 × 600 mg p.o. GEM (PPAR-α)	Double-blind, within-subjects, counterbalanced, placebo-controlled design. Two 2-week phases separated by 1-week washout period. During the first week, participants smoked normally, and laboratory measures of cue-elicited craving and forced-choice paradigms were taken. During the second week, participants were instructed to stop smoking, and abstinence was assessed.	GEM did not increase number of days of self-reported abstinence compared to placebo GEM had no impact on subjective/physiological reaction to smoking cues or reinforcing effects of nicotine (forced choice paradigm)
Jones et al., 2018 [47]	Non-treatment-seeking adults with opioid dependence, N = 30 (14 active, 16 placebo; 28 M, 2 F), 21–55 years old (mean 42.4 years in active group, 44.5 years in placebo group)	45 mg p.o. PIO (PPAR-γ)	Single-blind, between-subjects, randomized placebo-controlled design. Participants received PIO daily for 3 weeks. Laboratory testing (reinforcing effects, cue reactivity, subjective effects, cognitive effects, and physiological effects) began after the first week of buprenorphine/naloxone stabilization.	PIO did not influence the reinforcing effects of heroin (verbal choice SA or progressive choice paradigms) or physiological/subjective reactivity to active drug cues PIO did not influence the positive subjective effects of heroin PIO did further attenuate self-report ratings of anxiety during heroin self-administration, but had no impact on any other negative subjective effects PIO reduced ratings of “I want heroin”
Schroeder et al., 2018 [48]	Opioid-dependent adults undergoing a buprenorphine taper, N = 21 randomized (8 active, 13 placebo; 15 M, 6 F), N = 17 received at least one dose (6 active, 11 placebo), 18–65 years old (mean 38.4 years of participants randomized to active, 39.5 years placebo)	15 or 45 mg p.o. PIO (PPAR-γ)	Randomized, between-subjects design. Initial outpatient design (12 weeks of PIO treatment following 1-week buprenorphine stabilization), then subsequent outpatient/inpatient combination (5 weeks of PIO treatment following buprenorphine stabilization). Measures of opiate withdrawal collected daily throughout the study.	PIO significantly increased scores on the SOWS during the taper and post-taper phases, and had no effect on COWS scores In addition, there was no effect of PIO on opioid-positive urine samples during the post-taper phase

COWS, Clinical Opiate Withdrawal Scale; FEN, fenofibrate; GEM, gemfibrozil; p.o., per os (oral); PIO, pioglitazone; SA, self-administration; SOWS, Subjective Opiate Withdrawal Scale.
